# Association between HbA1c and carotid atherosclerosis among elderly Koreans with normal fasting glucose

**DOI:** 10.1371/journal.pone.0171761

**Published:** 2017-02-08

**Authors:** Seung Won Lee, Hyeon Chang Kim, Yong-ho Lee, Bo Mi Song, Hansol Choi, Ji Hye Park, Yumie Rhee, Chang Oh Kim

**Affiliations:** 1 Department of Public Health, Yonsei University Graduate School, Seoul, Republic of Korea; 2 Cardiovascular and Metabolic Diseases Etiology Research Center, Yonsei University College of Medicine, Seoul, Korea; 3 Department of Preventive Medicine, Yonsei University College of Medicine, Seoul, Korea; 4 Department of Internal Medicine, Yonsei University College of Medicine, Seoul, Korea; University of Colorado Denver School of Medicine, UNITED STATES

## Abstract

**Aim:**

We examined whether glycated haemoglobin (HbA1c) is associated to carotid atherosclerosis in an elderly Korean population with normal fasting glucose.

**Methods:**

Using data from the Korean Urban Rural Elderly study, we conducted a cross-sectional analysis of 1,133 participants (335 men and 798 women) with a mean age of 71.8 years. All participants had fasting blood glucose less than 100mg/dL (5.6 mmol/L) and HbA1c level below 6.5% (48 mmol/mol). They were also free from a history of cardiovascular disease, known type 2 diabetes mellitus or use of anti-diabetes medications. Carotid atherosclerosis was assessed by intima-media thickness (IMT) using ultrasonography. The association between HbA1c and carotid IMT was investigated using multivariable linear regression analysis.

**Results:**

HbA1c levels were independently and positively associated with carotid IMT (*β* = 0.020, *p* = 0.045) after adjusting for sex, age, body mass index, systolic blood pressure, diastolic blood pressure, triglyceride, LDL cholesterol, smoking and alcohol intake. However, fasting insulin and glucose levels were not associated with carotid IMT.

**Conclusion:**

HbA1c levels were positively associated with carotid atherosclerosis, as assessed by carotid IMT, in an elderly population with normoglycemia. Our study suggested that higher HbA1c level is an effective and informative marker of carotid atherosclerosis in an elderly population.

## Introduction

Glycated hemoglobin (HbA1c) is an well-known indicator used to assess long-term control of diabetes, and HbA1c level has been used as a diagnostic or screening tool for type 2 diabetes mellitus[1]. Previous studies showed positive associations of HbA1c levels with cardiovascular disease and mortality in those with diabetes mellitus[2–5]. Microvascular complications, such as nephropathy and retinopathy, are also associated with HbA1c[6]. Studies also observed a relationship between HbA1c and atherosclerosis among people with and without diabetes[7–9]. Carotid intima-media thickness (IMT) is known as a highly reproducible and non-invasive method to access atherosclerotic process[10–12]. Increased carotid IMT is also a predictor of atherosclerotic cardiovascular disease such as stroke and myocardial infarction[13–16]. Previous intervention studies reported that there were reductions of carotid IMT in patients with lipid-lowering drugs compared with a placebo group[17,18]. In people with diagnosed and unrecognized diabetes, HbA1c was independently related to carotid IMT[9]. Interestingly, HbA1c levels were positively associated with carotid IMT in the people with normoglycemia as well[7,8]. However, the association was not clear in the Asian population[7,8]. In an elderly Asian population with normal fasting glucose levels, the association between HbA1c and subclinical carotid atherosclerosis has not been fully investigated. Thus, in the current study, we evaluated the association between HbA1c and carotid IMT in an elderly Korean population with normal fasting glucose.

## Materials and methods

### Study population

This study analyzed baseline data in 2012 and 2013 from the Korean Urban Rural Elderly (KURE) study which is a community-based cohort study. The procedures of data collection in KURE have been described elsewhere in detail [19,20]. During the summers (June to September) of 2012 and 2013, a total of 2,025 participants aged more than 65 years were enrolled from urban and rural communities. All participants conducted questionnaire interview and health examination as a prescribed protocol. Among the 2,025 participants, based on the results of physical examination, people who had a fasting glucose level < 100 mg/dL (5.6 mmol/L) and an HbA1c level < 6.5% (48 mmol/mol) were included in the current analysis. Those with a history of major cardiovascular disease such as myocardial infarction or stroke (n = 273) or who were under treatment for diabetes (n = 349) were excluded from the current analysis. People who had missing important covariate data (n = 5) were also excluded. Finally, a total of 1,133 participants were included in the current analysis. All participants provided written informed consent forms and study protocol was approved by the Institutional Review Board of Severance Hospital at Yonsei University College of Medicine (approval number: 4-2012-0172; approval date: May 3, 2012). at the beginning of the study.

### Questionnaire data

The KURE study collected information on demographics, medical history, and lifestyle using standardized questionnaires. All interviewers were trained and performed the questionnaire surveys according to the prescribed procedure. Smoking status of participants was categorized as current smokers or current nonsmokers. Alcohol intake was classified in the current study as regular alcohol consumption (once a week or more) or other (less than once a week).

### Physical examination

The methods for physical examination in the KURE were previously reported in detail elsewhere[19,21,22]. All participants wore identical hospital gown for physical examination. A trained research examiner made anthropometric measurements including standing height (cm), body weight (kg) and waist circumferences as described previously and body mass index (BMI) was calculated [23]. Resting systolic and diastolic blood pressures (SBP and DBP) were measured more than twice using automatic sphygmomanometer (Omron HEM-7111, Omron Healthcare, Kyoto, Japan). Prior to each measurement, participants had rested for at least five minutes in sitting position. If the first and second measurements differ by ≥ 10 mmHg for either SBP or DBP, an additional measurement was performed, and the average of the last two measurements was used for analysis. Carotid IMT were measured by high-resolution B-mode ultrasonography using an 8-MHz linear probe (L5-13IS, Samsung Medicine, Seoul, Korea) in both carotid arteries. Both left and right carotid arteries were examined at levels of the distal common carotid artery, 1.0 cm proximal to the carotid bulb, and the proximal internal carotid artery in both transverse and longitudinal orientations by an single technician as a prescribed protocol[24]. The IMT was defined as the distance between the adventitia-media interface and intima-lumen interface[19]. IMT measurements were performed at all three segments providing a total of 6 measurements per person. The maximum value of the 6 measurements from the bilateral IMTs was used in the analysis. All scans were digitally stored for subsequent offline analysis. One end-diastolic frame (captured adjacent to the R wave on a continuously recorded ECG) for each interrogation angle was selected and analyzed for mean and maximum IMT. Carotid IMT was analyzed as markers of carotid atherosclerosis. We reported previously that inter-rater reliability of carotid IMT in our clinical centers. The inter-rater reliability of carotid IMT measurements among three clinical centers turned out to be high (calculated intraclass correlation coefficient 0.647 [95% CI: 0.487–0.779] for maximum carotid IMT)[25].

### Laboratory assays

After at least eight hours of fast, blood samples were collected from the antecubital vein in the morning. Fasting blood glucose level was determined by a colorimetry method (ADVIA 1800, Siemens, USA). Insulin level was measured by a chemiluminescence immunoassay (Centaur XP, Siemens, USA). Homeostasis model assessment of insulin resistance (HOMA-IR) was used to evaluate insulin resistance: HOMA-IR = fasting plasma glucose (mg/dL) × fasting insulin (μIU/mL)/405[26]. An enzymatic method (ADVIA 1800, Siemens, USA) was used to assess total cholesterol, high-density lipoprotein (HDL) cholesterol and triglycerides. C-reactive protein (CRP) concentration was assessed by a turbidometric immunoassay (ADVIA 1800, Wako, Japan). Low-density lipoprotein (LDL) cholesterol was calculated according to the Friedewald formula: LDL cholesterol = Total cholesterol—HDL cholesterol—(triglycerides/5) (all concentrations in mg/dL)[27]. HbA1c levels were measured by high-performance liquid chromatography using the VARIANT II system (Bio-Rad, Hercules, CA, USA).

### Statistical analysis

Gender differences in general characteristics were assessed by an independent t-test, Wilcoxon rank sum test, or chi-square test. Fasting insulin, HOMA-IR and CRP levels were log-transformed for parametric testing due to a right-skewed distribution. The relationships between interesting variables and other variables were evaluated using Pearson correlation analysis along with scatter plots.

In order to investigate an independent association between HbA1c and carotid IMT, we used multivariable linear regression analyses. Confounders were selected based on previous studies: sex, age, waist circumference, SBP, DBP, triglycerides, LDL cholesterol, HOMA-IR, smoking status, and alcohol intake. We also tested for the presence of effect modification by sex including interaction term in separate models. All analyses were performed using SAS statistical software, version 9.4 (SAS Institute, Cary, NC, USA), and all statistical tests were two-sided and *p* value less than 0.05 was considered significant.

## Results

The general characteristics of the study population are shown in Table 1. In this study, a total of 1,133 participants with a mean age of 71.8 years, consisting of 335 men and 798 women, were included. The mean HbA1c level was 5.5% (37 mmol/mol) and mean carotid IMT was 0.9 mm. Men tended to have a higher SBP, DBP, and waist circumference. However, men also had higher levels of HbA1c, insulin, lipid profiles, HOMA IR and CRP than women.

**Table 1:** General characteristics of the study population.

Variables	Total (n = 1,133)			Men (n = 335)			Women (n = 798)			*p* value^a^
Age, years	71.8	±	4.8	72.7	±	4.9	71.4	±	4.8	< .001
BMI, kg/m^2^	23.8	±	2.9	23.3	±	2.8	24.1	±	3.0	< .001
Waist circumference, cm	82.6	±	8.6	84.3	±	8.6	81.9	±	8.5	< .001
SBP, mmHg	127.2	±	15.2	128.6	±	14.5	126.7	±	15.4	0.044
DBP, mmHg	72.9	±	8.4	73.8	±	8.5	72.5	±	8.4	0.015
Carotid IMT, mm	0.9	±	0.1	0.9	±	0.1	0.8	±	0.1	< .001
Fasting glucose, mg/dL	88.5	±	6.2	88.4	±	6.4	88.6	±	6.1	0.646
Fasting insulin, mg/dL	5.1	[3.7–7.5]		4.3	[3.1–6.3]		5.5	[3.9–8.0]		0.007
HbA1c, %	5.5	±	0.3	5.4	±	0.3	5.5	±	0.3	0.028
Total cholesterol, mg/dL	188.5	±	34.7	178.7	±	30.3	192.6	±	35.7	0.292
HDL cholesterol, mg/dL	51.7	±	12.3	48.2	±	11.2	53.1	±	12.4	0.003
LDL cholesterol, mg/dL	111.6	±	30.2	106.3	±	27.0	113.9	±	31.3	< .001
Triglyceride, mg/dL	126.0	±	61.1	121.3	±	58.1	127.9	±	62.2	< .001
HOMA-IR	1.1	[0.8–1.7]		0.9	[0.7–1.4]		1.2	[0.8–1.8]		< .001
CRP, mg/L	0.7	[0.4–1.4]		0.8	[0.4–1.5]		0.7	[0.4–1.4]		< .001
Smoking, current	72	(6.4)		59	(17.7)		13	(1.6)		< .001
Alcohol intake, current	394	(34.8)		186	(55.7)		208	(26.1)		< .001

Values are presented as mean ± standard deviation, median [interquartile range], or number (%).

^a^*P* values for comparison of gender characteristics are based independent t-test, the Wilcoxon rank sum test, or chi-square test.

*P* values compare men and women using independent t-test, the Wilcoxon rank sum test, or chi-square test.

Abbreviations: BMI, body mass index; CRP, C-reative protein; DBP, diastolic blood pressure; HDL, high-density lipoprotein; HOMA-IR, homeostasis model assessment of insulin resistance; IMT, intima-media thickness; LDL, low-density lipoprotein; SBP, systolic blood pressure

**Fig 1:**
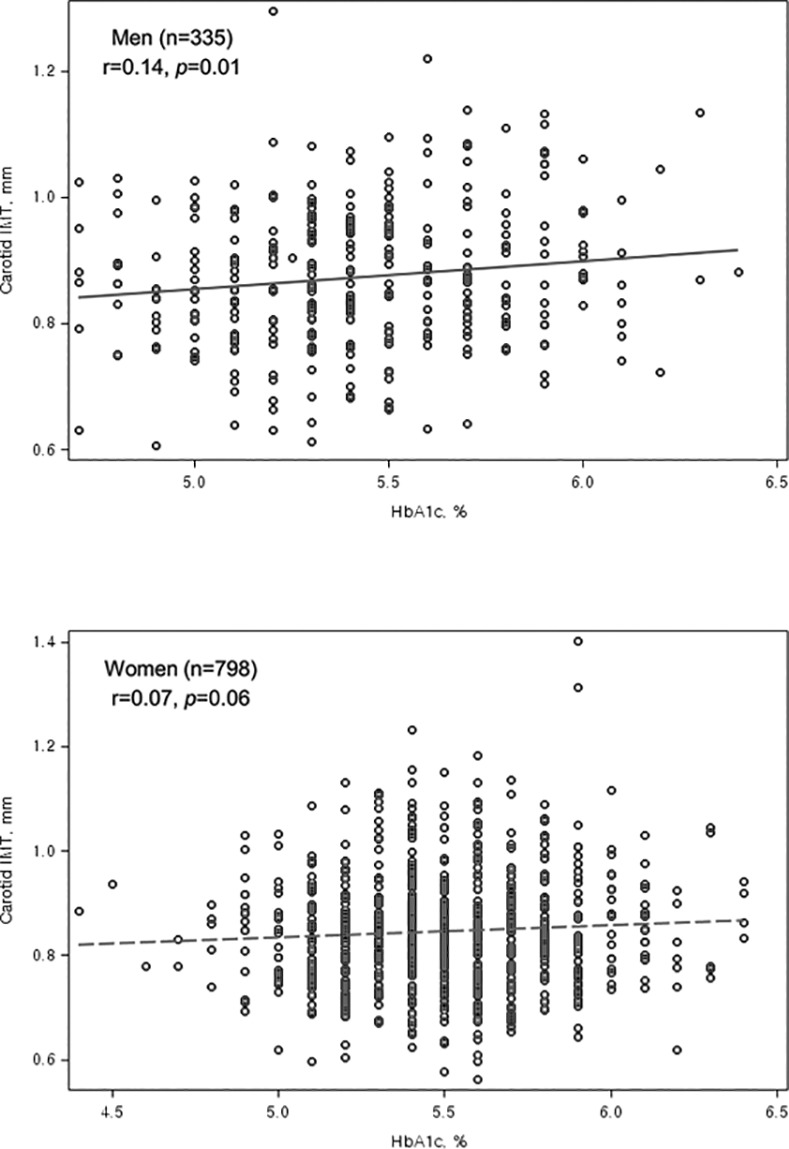
Pearson correlation coefficient and *p* value between carotid IMT and HbA1c.

Table 2 shows the correlations of carotid IMT and other variables. HbA1c was significantly and positively correlated with carotid IMT after adjusting for sex and age. Conversely, fasting glucose and insulin levels were not associated with carotid IMT after adjusting for age. The unadjusted association between HbA1c and carotid IMT is also showed by scatter plots, separately for men and women (Fig 1). The positive association between HbA1c and carotid IMT was more prominent in men than in women.

**Table 2:** Correlation of carotid IMT with other variables.

	Simple Correlation		Partial Correlation^a^	
Total (n = 1,133)	Pearson coefficients	*p* value	Pearson coefficients	*p* value
Age	0.168	< .001		
BMI	0.081	0.006	0.110	< .001
Waist circumference	0.135	< .001	0.120	< .001
SBP	0.132	< .001	0.104	< .001
DBP	- 0.019	0.527	- 0.011	0.711
HbA1c	0.077	0.009	0.084	0.005
Fasting glucose	- 0.014	0.644	- 0.015	0.623
Fasting insulin (logarithmic)	- 0.007	0.820	0.019	0.514
Total cholesterol	0.053	0.075	0.081	0.006
HDL cholesterol	- 0.123	< .001	- 0.105	< .001
LDL cholesterol	0.080	0.007	0.102	< .001
Triglycerides	0.076	0.011	0.079	0.008
HOMA-IR (logarithmic)	- 0.008	0.794	0.017	0.561
CRP (logarithmic)	0.058	0.049	0.049	0.098
Smoking	0.037	0.208	< .001	0.996
Alcohol intake	- 0.002	0.930	- 0.018	0.541

^a^Partial correlation analysis with adjustment for sex and age.

Abbreviations: BMI, body mass index; CRP, C-reative protein; DBP, diastolic blood pressure; HDL, high-density lipoprotein; HOMA-IR, homeostasis model assessment of insulin resistance; IMT, intima-media thickness; LDL, low-density lipoprotein; SBP, systolic blood pressure.

Table 3 shows the relationship between HbA1c and carotid IMT in multivariable linear regression models. HbA1c was positively associated with carotid IMT (*β* = 0.026, *p* = 0.009). The positive association between HbA1c and carotid IMT was not affected by adjustment for sex, body mass index (BMI), SBP, DBP, triglyceride, LDL cholesterol, HOMA-IR, smoking, and alcohol consumption (*β* = 0.020, *p* = 0.045). The interactions between sex and HbA1c for carotid IMT was not significant (*p* = 0.3374). However, when examining this association for men and women separately, the association between HbA1c and carotid IMT was not significant in women.

**Table 3:** Association between HbA1c and carotid IMT.

	Unadjusted				Adjusted^a^		
	*β*	SE	R^2^	*p* value	*β*	SE	*p* value
Total (n = 1,133)							
Sex, male	- 0.027	0.007	0.013	< .001	- 0.031	0.008	< .001
Age, 10 years	0.038	0.007	0.028	< .001	0.025	0.007	< .001
BMI, kg/m^2^	0.003	0.001	0.007	0.006	0.005	0.001	< .001
HbA1c, %	0.026	0.010	0.006	0.009	0.020	0.010	0.045
SBP, 10 mmHg	0.009	0.002	0.018	< .001	0.017	0.003	< .001
DBP, 5 mmHg	- 0.001	0.002	< .001	0.527	- 0.013	0.003	< .001
Triglyceride, g/dL	0.135	0.053	0.006	0.011	0.113	0.054	0.035
LDL cholesterol, g/dL	0.289	0.107	0.006	0.007	0.345	0.104	0.001
HOMA-IR (logarithmic)	< .001	0.006	< .001	0.955	- 0.007	0.006	0.093
CRP, mg/L (logarithmic)	0.006	0.003	0.003	0.049	0.002	0.003	0.463
Current smoking	- 0.019	0.006	0.010	0.001	< .001	0.014	0.979
Current drinking	- 0.001	0.007	< .001	0.930	- 0.003	0.007	0.711
					Adjusted R^2^ = 0.0868		
Men (n = 335)							
Age, 10 years	0.045	0.012	0.041	< .001	0.041	0.013	0.002
BMI, kg/m^2^	0.004	0.002	0.010	0.065	0.006	0.003	0.008
HbA1c, %	0.044	0.017	0.019	0.012	0.036	0.017	0.040
SBP, 10 mmHg	0.004	0.004	0.002	0.371	0.010	0.005	0.076
DBP, 5 mmHg	- 0.007	0.004	0.012	0.049	- 0.011	0.005	0.023
Triglyceride, g/dL	0.128	0.104	0.005	0.217	0.095	0.108	0.372
LDL cholesterol, g/dL	0.170	0.224	0.002	0.449	0.149	0.220	0.497
HOMA-IR (logarithmic)	- 0.002	0.010	< .001	0.814	- 0.016	0.012	0.070
CRP, mg/L (logarithmic)	0.002	0.006	< .001	0.765	-0.001	0.006	0.861
Current smoking	- 0.005	0.016	< .001	0.756	-0.005	0.016	0.753
Current drinking	- 0.029	0.012	0.017	0.018	-0.020	0.012	0.094
					Adjusted R^2^ = 0.0797		
Women (n = 798)							
Age, 10 years	0.030	0.008	0.018	< .001	0.016	0.008	0.048
BMI, kg/m^2^	0.003	0.001	0.009	0.008	0.004	0.001	0.002
HbA1c, %	0.023	0.012	0.005	0.058	0.013	0.012	0.303
SBP, 10 mmHg	0.011	0.002	0.025	< .001	0.020	0.004	< .001
DBP, 5 mmHg	0.001	0.002	< .001	0.821	- 0.014	0.003	< .001
Triglyceride, g/dL	0.152	0.061	0.008	0.013	0.120	0.062	0.051
LDL cholesterol, g/dL	0.392	0.121	0.013	0.001	0.406	0.118	0.001
HOMA-IR (logarithmic)	0.003	0.007	0.001	0.447	- 0.004	0.008	0.408
CRP, mg/L (logarithmic)	0.008	0.004	0.005	0.049	0.005	0.004	0.215
Current smoking	0.026	0.030	0.001	0.381	0.032	0.029	0.277
Current drinking	0.002	0.009	< .001	0.815	0.008	0.008	0.370
					Adjusted R^2^ = 0.0771		

^a^Adjusted for other variables in the table.

Abbreviations: BMI, body mass index; CRP, C-reative protein; DBP, diastolic blood pressure; HOMA-IR, homeostasis model assessment of insulin resistance; IMT, intima-media thickness; LDL, low-density lipoprotein; SBP, systolic blood pressure; SE, standard error.

## Discussion

We investigated the association between HbA1c and carotid IMT in among elderly Koreans with a normal fasting glucose. HbA1c was positively related to carotid IMT. The association between HbA1c and carotid IMT was significant before and after adjustment for covariates, particularly in men. Although there are a significant relationship between HbA1c and carotid IMT only for men in our study, there is a significant relationship between HbA1c and carotid IMT in total population and the interaction test for sex was not significant (*p* = 0.3374). In our study, carotid IMT was defined by maximum thickness value. The average thickness value was not significantly associated with HbA1c as well as fasting glucose and insulin contrary to maximum thickness value in our study. We could not elucidate the reason, there is a possibility linking the characteristics of study population. Our study population was relatively healthy and without major cardiovascular disease such as myocardial infarction or stroke and diabetes mellitus. They might be still early stages of carotid atherosclerosis.

Previous researches have investigated the association between HbA1c and atherosclerosis assessed by carotid IMT in persons without diabetes [8,28–30]. A positive association was reported in several cross-sectional studies on diverse ethnic groups, including American, German, Chinese, and Asian Indians[7,8,28,31]. A consistent association was also observed in our study participants who were elderly and non-obese. Previous studies analyzed relatively young (40–60 years) and obese (BMI > 25 kg/m^2^) participants, unique from our patient pool[7,8,28,31]. However, a previous Korean study among people without diabetes HbA1c was not associated with carotid IMT and plaque, but HbA1c was positively associated with brachial-ankle pulse wave velocity[32]. A previous prospective study also reported that HbA1c was not related to carotid atherosclerosis[29]. This study included people with a < 110 mg/dL (6.1 mmol/L) fasting glucose level, whereas our study included people with fasting blood glucose less than 100 mg/dL (5.6 mmol/L) and HbA1c level below 6.5% (48 mmol/mol). According to our analyses, fasting glucose and insulin were not associated with carotid IMT in elderly population with normal fasting glucose. This finding may be attributed to the characteristics of our study subjects, whose average HOMA-IR was 1.1 [0.8–1.7], indicating a highly insulin sensitive condition. This value was much lower than that reported by Venkataraman et al. (HOMA-IR of 1.4 to 1.9)[28], despite a similar BMI distribution. In a previous study with 582 individuals aged 40–70 years without known diabetes, post-challenge plasma glucose presented a strong association with atherosclerosis, but fasting glucose and HbA1c levels did not have a significant association with atherosclerosis[30]. On the other hand, HbA1c had positive and significant associations with carotid IMT in our study. In a previous Korean study of diabetic patients, HbA1c was positively related to carotid plaques, but not with carotid IMT[33]. This previous study examined association between HbA1c, carotid atherosclerosis, arterial stiffness, and peripheral arterial disease among Korean patients with type 2 diabetes (n = 370) registered with the public health center. But we examined HbA1c and carotid IMT using a larger sample with relatively healthy community dweller.

Recent studies have shown that hyperglycemia may lead to arteriopathy by inducing oxidative stress, which facilitate the formation of advanced glycosylation end products[34]. Glycosylation end products are associated with glycation and susceptibility to LDL oxidation and subsequent uptake by macrophages, thereby creating foam cells[35], which is associated with atherosclerosis initiation. Macrophages are important factors in all atherosclerotic stages[36]. In diabetic patients, approximately 80% of deaths is caused by atherosclerosis[34]. Hyperglycemia is a well-known pathogenic factor of atherosclerosis as it leads to alteration of vascular tissue at the cellular level and consequent atherosclerotic disease[34]. In diabetes, these alterations are caused by nonenzymatic glycosylation of proteins and lipids. This glycosylation can cause dysfunction of protein and lipid by obstructing enzymatic activity and receptor recognition[37]. Furthermore, glycosylated proteins were associated with a specific receptor of cells related to the atherosclerotic process such as monocyte-derived macrophages, endothelial cells, and smooth muscle cells. Their interaction can increase production of oxidative stress and proinflammatory responses that promote the atherosclerotic process[34].

The mechanism linking HbA1c and carotid atherosclerosis has still not been fully elucidated. However, some potential mechanisms that may be responsible for these associations have been suggested. People who had a relatively higher level of fasting glucose within the normal range may have higher postprandial glucose levels, which may be reflected by HbA1c[38]. Furthermore, postprandial glucose levels are more closely associated with increased carotid atherosclerosis[39,40]. Moreover, even among people with normal fasting glucose, people who had a glycometabolism disorder were at risk for increased carotid atherosclerosis[41,42]. Although we could not verify this possibility because we did not measure post-prandial glucose levels, HbA1c can reflect glucose excursions after a meal. HbA1c could be a better indicator of glycometabolism disorder, because measurement errors and intra-individual variability are less than for fasting glucose and insulin. In addition to, current smoking and alcohol intake were more prevalent among men than women in our study. Previous studies reported that smoking may increase the risk of cardiovascular heart disease by promoting atherosclerosis progression[43] suggesting that enhanced inflammation and excessive vascular remodelling caused by smoking are contributing factors in the development of accelerated atherosclerosis[44].

Our findings suggest that the atherosclerotic process is common in people with normal fasting glucose levels, and a higher HbA1c may increase the risk of carotid atherosclerosis. Some previous studies reported that glycemic control, as reflected by level of HbA1c, was related to carotid IMT among people with diabetes[9].

Our study does have some limitations. First, the current study has limitation due to its cross-sectional design. It was not possible to explain the causal relationship between HbA1c levels and carotid atherosclerosis. Second, it may not be appropriate to generalize it to another ethnic or age group because the study was performed with elderly Koreans. Third, as we could not conduct an oral glucose tolerance test, participants with diabetes or impaired fasting glucose could be misclassified as normal. Misclassification, if any, would be expected to bias our study results toward the null value. Thus, our study showed probably conservative results, and null findings of the previous studies can be partially explained by this potential misclassification.

Nevertheless, our study has several distinct strengths. The study population consists of rather homogenous and large numbered elderly people. In addition, we focused on elderly people in a metabolically healthy condition for the purpose of investigating the association between HbA1c and carotid IMT. As the study population was rather lean, insulin-sensitive and had lower blood pressure and LDL levels, we can exclude the effect of these confounding factors that can affect the progression of atherosclerosis.

## Conclusions

Our study showed that increased HbA1c levels are positively related to carotid atherosclerosis assessed by carotid IMT in an aged people with normoglycemia. Atherosclerotic risk seemed to increase linearly according to HbA1c levels, even within normoglycemic individuals. HbA1c level within the normal fasting glucose range may serve as a subclinical marker of carotid atherosclerosis in an elderly population. Fasting glucose is insufficient to verify atherosclerotic cardiovascular disease risk in an elderly population based on our study. Post-prandial glucose testing is difficult to carry out, especially in an elderly population, and requires more time and cost than a simple HbA1c test. In an elderly population, HbA1c level is a more effective and informative marker for high-risk atherosclerotic cardiovascular disease screening.

## Supporting information

S1 Table Minimal data set. (XLSX)

## Abbreviations

BMI: body mass index
CRP: C-reactive protein
DBP: diastolic blood pressure
HOMA-IR: homeostasis model assessment of insulin resistance
HDL: high-density lipoprotein
IMT: intima-media thickness
KURE: Korean Urban Rural Elderly
LDL: Low-density lipoprotein
SBP: Systolic blood pressure
